# Genetic diversity and virulence of novel sequence types of *Streptococcus suis* from diseased and healthy pigs in China

**DOI:** 10.3389/fmicb.2015.00173

**Published:** 2015-03-02

**Authors:** Shujie Wang, Mingming Gao, Tongqing An, Yonggang Liu, Jiamin Jin, Gang Wang, Chenggang Jiang, Yabin Tu, Shouping Hu, Jinsong Li, Jie Wang, Dongsheng Zhou, Xuehui Cai

**Affiliations:** ^1^State Key Laboratory of Veterinary Biotechnology, Harbin Veterinary Research Institute – Chinese Academy of Agricultural SciencesHarbin, China; ^2^State Key Laboratory of Pathogen and Biosecurity, Beijing Institute of Microbiology and EpidemiologyBeijing, China

**Keywords:** *Streptococcus suis*, multilocus sequence typing, virulence

## Abstract

*Streptococcus suis* is a serious threat to swine industry and public health. In this work, a total of 62 *S. suis* isolates recovered from infected and healthy pigs from four provinces in northern China were classified by multilocus sequence typing into nine sequence types (STs), including six novel ones, namely, ST417, ST418, ST419, ST420, ST421, and ST422. The majority (64.5%) of these 62 isolates belong to serotype 2; all of these serotype 2 isolates can be assigned into ST1 or ST28 clonal complex, indicating at least two parallel routes of clonal dissemination of these isolates. In these serotype 2 isolates, 23 (20 from healthy pigs and three from diseased pigs) were identified as ST7 strains, which were previously characterized as the cause of streptococcal toxic shock-like syndrome. The novel ST strains lack 89 K pathogenicity island but can cause septicemia and meningitis in a mouse model, showing remarkable differences in virulence. The ST421 strain named HLB causes suppurative encephalitis. Our results highlighted the need for increased surveillance of *S. suis* in farm-raised pigs in northern China.

## INTRODUCTION

*Streptococcus suis* is an important pathogen of pigs and one of the most important causes of bacterial mortality in post-weaning piglets (Feng et al., 2014). At least 33 capsular serotypes (1 to 31, 33, and 1/2) of *S. suis* have been described; among highly virulent strains, serotype 2 is the most dominant (Higgins and Gottschalk, 1995; Liu et al., 2013). Meningitis is the most striking feature of *S. suis* infection in pigs; the presence of fibrin, edema, and cellular infiltrates in meninges and choroid plexus, along with adjacent encephalitis, are commonly observed histopathological characteristics (Madsen et al., 2002). Animal models of *S. suis*-induced meningitis have been widely characterized with serotype 2; however, other serotypes have been rarely used (Fittipaldi et al., 2011).

*Streptococcus suis* can cause meningitis, septicemia, endocarditis, and arthritis in humans in close contact with infected pigs or pork-derived products. In China, *S. suis* serotype 2 causes two outbreaks of human infection, which is characterized as streptococcal toxic shock-like syndrome (STSS) with higher-than-usual morbidity and mortality (Tang et al., 2006; Ye et al., 2006; Yu et al., 2006; Feng et al., 2014). STSS-causing *S. suis* has evolved to acquire, most likely through horizontal gene transfer, an 89 K pathogenicity island (89 K PaI) with multiple virulence genes (Zhu et al., 2008; Segura, 2009; Li et al., 2011). 89 K PaI-negative *S. suis* can also affect humans and cause fatal infections (Feng et al., 2014). *S. suis* contains an array of virulence genes, including those that encode outer membrane proteins, extracellular proteases, transporters, and secretion systems and their effectors, which are located inside and outside 89 K PaI (Feng et al., 2014).

Multilocus sequence typing (MLST), a highly discriminatory method used to characterize bacterial population structure (Chan et al., 2001; Adiri et al., 2003; Fearnhead et al., 2005), has been performed to investigate genotypes and microevolution of *S. suis* since 2002 (King et al., 2002; Wang et al., 2008). Thus far, 1,415 *S. suis* strains have been recorded in the *S. suis* MLST database ^1^; these strains can be classified into 616 STs in particular including 47 STs (corresponding to 218 isolates) from China.

In northern China, *S. suis*-associated diseases in farm-raised pigs typically occur from the end of October to the middle of January of the succeeding year; at these times, *S. suis* strains can be steadily isolated from infected pigs. This study aimed to characterize genetic diversity, microevolution, and virulence of *S. suis* isolates from the farm-raised pigs in northern China.

## MATERIALS AND METHODS

### BACTERIAL STRAINS

A total of 62 *S. suis* strains were isolated from different individuals of healthy or diseased pigs in swine farms in four provinces, namely, Heilongjiang, Jilin, Liaoning, and Hebei, in northern China from March 2007 to the autumn of 2010. *S. suis*-specific glutamate dehydrogenase (*gdh*) gene was targeted for PCR to identify this bacterial species (Okwumabua et al., 2003). *S. suis* capsular types were discriminated by using *S. suis* antisera specific for individual known serotypes from the Statens Serum Institute (SSI, Denmark); the distinguished types were then confirmed with a co-agglutination test, as previously reported (Higgins and Gottschalk, 1990).

### MULTILOCUS SEQUENCE TYPING

Bacteria were grown overnight at 37°C in Todd-Hewitt Broth (THB) and genomic DNA was then isolated using a TIANamp bacterial DNA kit (TIANGEN, China). For MLST (King et al., 2002), the DNA fragments of seven housekeeping genes, including *aroA*, *cpn60*, *dpr*, *gki*, *mutS*, *recA*, and *thrA*, were amplified by PCR. Both DNA strands were sequenced with PCR primers on ABI-3700 sequencer. DNA sequences were aligned using MUSCLE Version 3.8 (Edgar, 2004). The alleles and the ST number of each isolate were determined by querying the *S. suis* MLST database ^2^. Clonal complexes were identified and the overall population structure was determined using the eBURST software (Feil et al., 2004). Two different STs sharing six of the seven loci constituted a single-locus variant (SLV). A double-locus variant (DLV) contained two STs that differed in two loci but share the same other loci. A triple-locus variant (TLV) included two STs that differed in three loci. A clonal complex was composed of at least three STs with only SLVs. Two STs belonging to the same group with SLV were called a doublet. The remaining STs that did not contain SLV with other STs were termed singletons. The founders (ancestry types) of CCs were predicted with 1,000 re-samplings for bootstrap.

### DETECTION OF VIRULENCE MARKERS

Virulence markers encoding muramidase-released protein (MRP), extracellular protein factor (EF), suilysin (SLY), and 89 K PaI were detected by PCR, as previously described (Allen et al., 2001; Wisselink et al., 2002; Chen et al., 2007). *S. suis* strain 05ZYH33, a serotype 2 reference strain isolated from the human STSS case and harboring these virulence markers (Chen et al., 2007), was used as positive control in PCR.

### EXPERIMENTAL MOUSE INFECTION

Animal infection experiments were conducted following the guidelines and approved protocols of the Heilongjiang Province Experiment Animal Care and Use Committee. Each group of 16 female mice (6-weeks-old) was infected with one tested strain; bacterial suspension [1 ml; ∼5 × 10^7^ colony forming units (CFU)/ml] or sterile THB was administered intraperitoneally to each mouse (Dominguez-Punaro et al., 2007). The mice were monitored daily for 12 days to determine mortality rates and clinical signs. Blood (5 μl) was collected from the tail vein of each infected mouse at different days after infection; blood was then plated onto sheep blood agar plates for bacterial growth overnight at 37°C to evaluate bacterial loads (CFU/ml of blood). Bacterial load (CFU/0.05 g of organ specimens) in the liver or in the brain of each dead mouse at necropsy was also counted; organ specimens were subsequently fixed in 10% buffered formalin for histopathological analysis.

### STATISTICAL ANALYSIS

ANOVA was performed using GraphPad Prism (version 5.02 for Windows; GraphPad Software Incorporated, La Jolla, CA, USA).

## RESULTS

### GENETIC DIVERSITY

Among 62 *S. suis* isolates (**Table 1**), 36 were from healthy pigs and 26 were from diseased pigs. Among the 36 isolates from healthy pigs, five were from Hebei, six were from Heilongjiang, and 25 were from Jilin; conversely, the 26 isolates from diseased pigs were distributed among seven counties in Heilongjiang (**Table 1**). Furthermore, 54 of the 62 isolates could be assigned into serotypes 2 (40 isolates), 7 (nine isolates), and 9 (five isolates); however, the eight remaining isolates could not be typed on the basis of the antisera panel of the 33 known serotypes (**Table 1**).

**Table 1:** General characteristics of 62 *Streptococcus suis* isolates characterized in this study.

Number of strains (*n =* 62)	Serotype (*n =* 3)	Multilocus sequence typing (MLST) allelic profile (*n =* 9)	Sequence types (STs) (*n =* 9)	Clonal complex	Location (province–city)	Source
5	7	8,30,5,34,30,3,25	ST29	ST25	Hebei-SJZ	Healthy pigs, nose swabs, Autumn 2008
6	2	2,30,5,34,31,3,25	ST28	ST28	Heilongjiang-ZD	Healthy pigs, nose swabs, Autumn 2010
5	2	2,30,5,34,31,3,25	ST28		Jilin-DH	Healthy pigs, nose swabs, Spring 2007
3	2	1,30,5,34,31,4,25	ST418		Heilongjiang-JMS	Diseased pigs, brains, sporadic, Autumn 2007
20	2	1,1,1,1,1,1,3	ST7	ST1	Jilin-CC	Healthy pigs, nose swabs, Spring 2010
3	2	1,1,1,1,1,1,3	ST7		Heilongjiang-MDJ	Diseased pigs, synovia, sporadic, Autumn 2007
3	2	1,1,1,1,31,1,3	ST419		Heilongjiang-HD	Diseased pigs, brains, sporadic, Winter 2010
5	9	45,56,1,15,57,52,55	ST417	Not applicable	Heilongjiang-WC	Diseased pigs, brains, outbreak, Autumn 2007
4	Unknown	1,35,2,7,1,52,28	ST421		Heilongjiang-LB	Diseased pigs, brains, sporadic, Winter 2010
4	Unknown	7,58,36,54,42,37,12	ST422		Heilongjiang-HL	Diseased pigs, brains, sporadic, Winter 2010
4	7	7,58,36,54,42,34,12	ST420		Heilongjiang-JB	Diseased pigs, brains, sporadic, Winter 2010

**FIGURE 1:**
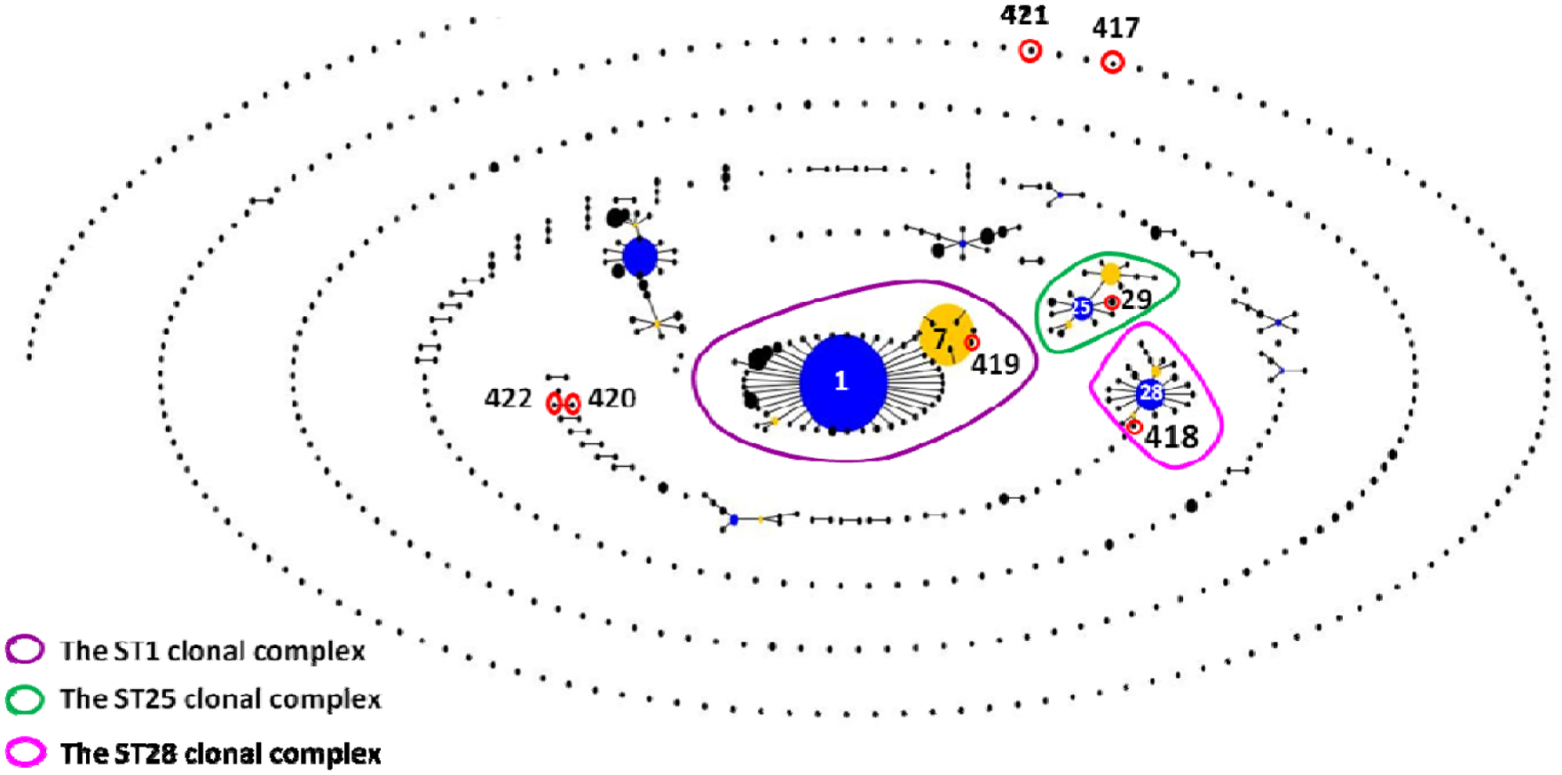
**eBURST-based snapshot of sequence types (STs).** Population snapshot of 1,415 *Streptococcus suis* strains (616 STs) from the Multilocus sequence typing (MLST) database was diagramed by eBURST on the basis of their allelic profiles. STs with single-locus variant (SLV) relationship were linked together to form various clonal complexes and doubletons. The size of the solid circle represented the number of isolates of each ST. The blue-highlighted solid circles (STs) represented the primary founders of the corresponding clonal complexes.

The 62 isolates (**Table 1**; **Figure 1**) could be assigned into three known STs [ST28 (11 isolates), ST29 (five isolates), and ST7 (23 isolates)] and six novel STs [ST417 (five isolates), ST418 (three isolates), ST419 (three isolates), ST420 (four isolates), ST421 (four isolates), and ST422 (four isolates). ST29 belonged to the ST25 complex, ST28, and ST418 belonged to the ST28 clonal complex, and ST7 and ST419 belonged to the ST1 complex; the other remaining STs identified in this study could not be assigned into any known clonal complex. ST420 contained SLV with ST422, and these two STs constituted a doublet group. The two singletons ST417 and ST421 did not show three or more allelic matches to any ST in the entire database; thus, these singletons could not be placed in any group by eBURST.

All of the isolates from healthy pigs belonged to ST25, ST28, and ST1 complexes. Among the 26 isolates from diseased pigs, six belonged to the ST1 complex (three to each of ST7 and ST419), three were assigned to ST418 of the ST28 complex, and 17 were classified into six novel STs. The six novel STs were found in the isolates from diseased pigs.

The 23 novel ST strains were selected and subjected to PCR-based screening of four virulence markers, namely, *mrp*, *sly*, *epf*, and 89 K PaI (**Table 2**). 89 K PaI and *mrp* were absent from these strains. *epf* was detected in ST418 and ST419 strains but not in the other strains. *sly* was found in ST418, ST419, and ST422 strains but not in the other strains. ST417, ST420, and ST421 strains did not contain any of these virulence markers.

**Table 2:** Prevalence of virulence markers.

Sequence types	Presence (+) or absence (-) of				Strain selected for mouse infection
	*mrp*	*sly*	*epf*	89 K PaI	
ST417	-	-	-	-	HWS9
ST418	-	+	+	-	HJMS
ST419	-	+	+	-	HDS
ST420	-	-	-	-	HJB
ST421	-	-	-	-	HLB
ST422	-	+	-	-	Not selected
ST335	+	-	-	-	HW07

### LETHALITY TO MICE

In mouse infection, one isolate was randomly selected from ST417 to ST420. Only one isolate from ST421 was included because ST421 and ST422 were unknown serotypes. The strains were coded as HWS9, HJMS, HDS, HJB, and HLB corresponding to ST417–ST421, respectively (**Table 2**). The ST335 strain HW07 (*mrp*^+^, *sly*^-^, *epf*^-^, and 89 K PaI^-^) is highly lethal to CD1 mice (Wang et al., 2013b); as such, this strain was used as a positive control of mouse infection.

**FIGURE 2:**
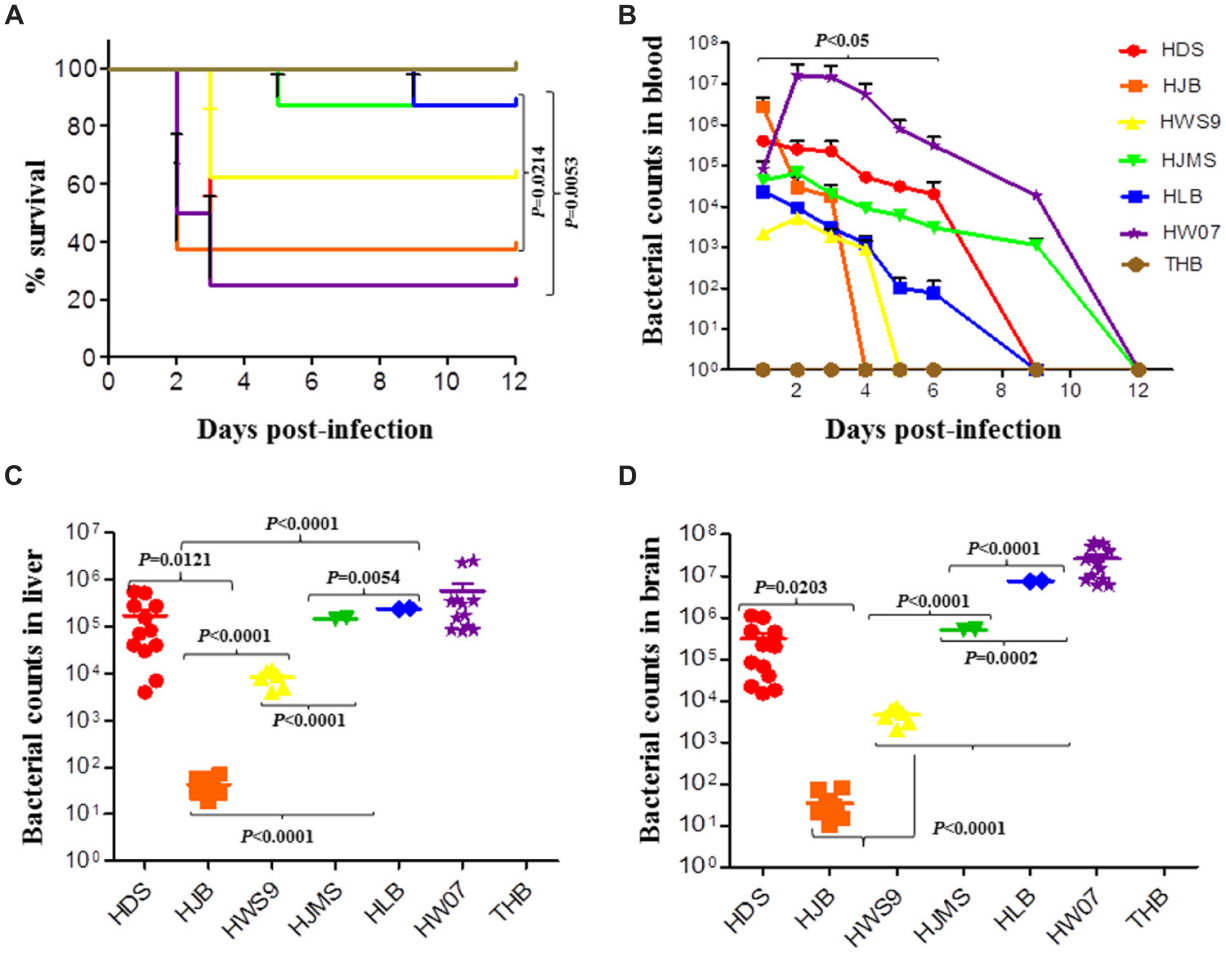
**Virulence of *S. suis* in the CD1 mouse model.** Mice were challenged with the indicated strains, as described in Experimental Procedure. **(A)** Survival rates of the CD1 mice inoculated with *S. suis*. **(B)** Bacterial loads in the blood (CFU/ml of blood); HDS and HLB are significantly different (*p* < 0.05). **(C)** Bacterial loads in the liver of mice (CFU/0.05 g of tissue). **(D)** Bacterial loads in the brain (CFU/0.05 g of tissue).

The mortality rates of HW07, HDS, HJB, HWS9, HJMS, and HLB at 12 days post-infection were 75, 75, 62.5, 37.5, 12.5, and 12.5%, respectively (**Figure 2A**). These six strains could be roughly divided into three groups based on mortality rates, the time at which clinical symptoms manifested, the degree of severity of clinical symptoms, and the time at which death occurred: high virulence group (HW07, HDS, and HJB); moderate virulence group (HWS9); and low virulence group (HJMS and HLB).

The high virulence group could be further divided into super acute death subgroup (HJB) and acute death subgroup (HW07 and HDS). The super acute death subgroup rapidly displayed clinical symptoms, such as neurasthenia and hind limb disorders, causing death as early as 1 day post-infection; by contrast, the acute death subgroup manifested clinical symptoms much later and death occurred at 2–3 days post-infection. In summary, all of the five novel ST strains evaluated with the positive control strain were virulent to mice but exhibited remarkable differences in lethality to mice.

### BACTERIAL COUNTS IN INFECTED MICE

The bacterial loads of the strains sharply increased and then gradually decreased in the blood of the infected mice (**Figure 2B**). The maximum bacterial load of the five novel ST strains in the blood was detected at 1 day post-infection; by contrast, the maximum bacterial load of the positive control strain HW07 was found at 2 days post-infection. Bacteria could be recovered from the blood until 4 days post-infection. These results indicated that all of the strains caused bacteremia in mice. The maximum bacterial load of the high virulence group (>5 × 10^5^ CFU/ml) was larger than that of the moderate virulence group (<1 × 10^4^ CFU/ml) and the low virulence group (∼5 × 10^4^ CFU/ml). Other contributors, in addition to bacteremia, might have caused the death of mice in the moderate virulence group because this group exhibited a smaller maximum bacterial load than the low virulence group.

Viable bacteria could be recovered from the liver (**Figure 2C**) and the brain (**Figure 2D**) of all the dead mice. In both organs, the HJB strain displayed very low bacterial load (50 CFU/ml) probably because this strain caused the death of mice very rapidly; as a result, this strain could not abundantly reproduce in the organs. By contrast, the other strains yielded bacterial loads larger than 5 × 10^3^ CFU/ml, indicating abundant bacterial proliferation in the organs as infection developed.

### MICROSCOPIC LESIONS OF DEAD MICE

**FIGURE 3:**
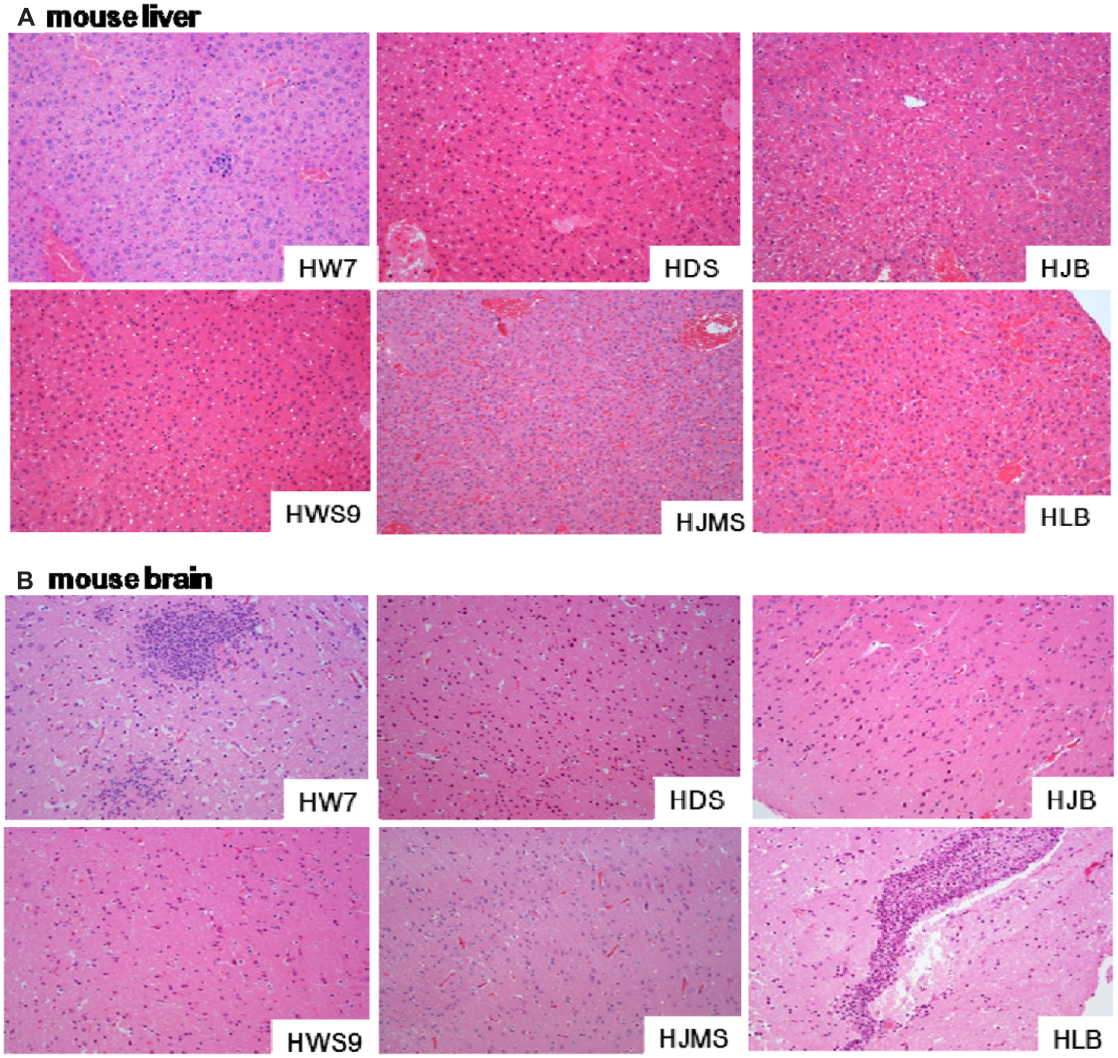
**Histopathological observations in tissues.** Liver **(A)** or brain **(B)** specimens from the dead mouse at necropsy were fixed in 10% buffered formalin for histopathological analysis. The tissues were stained with hematoxylin and eosin. Single representative image of each organ for each strain. Magnification, 100×.

Different strains caused different degrees of histopathological injury in the liver (**Figure 3A**) and in the brain (**Figure 3B**) of the dead mice. The damage in the liver was positively correlated with the lethality to mice (**Figure 3A**). The high virulence group (HW07, HDS, and HJB) caused severe liver cell inflammation, vacuolar degeneration, and inflammatory cell infiltration. The moderate virulence group (HWS9) led to evident liver cell inflammation accompanied by slight vacuolar degeneration and lymphocyte infiltration. The low virulence group (HJMS and HLB) caused only slight lymphocyte infiltration without liver cell inflammation and vacuolar degeneration.

All the tested strains could break through the blood–brain barrier (BBB), causing bacterial encephalitis. The severity of brain injury was positively correlated with the bacterial load in the brain (**Figure 3B**). HW07 and HLB caused the most severe brain injury, which is a typical characteristic of suppurative encephalitis with heavy neutrophil necrosis and collapse. HDS, HJMS, and HWS9 induced the production of numerous glial cells. HJB caused slight inflammatory response in the brains, as indicated by limited glial cell production.

## DISCUSSION

We isolated 62 *S. suis* strains from healthy and diseased pigs in the swine farms in northern China; among these strains, 40, 9, and 5 belonged to serotypes 2, 7, and 9, respectively; the eight remaining isolates had undetermined serotypes because the serum agglutination test with the 33 standard antisera of *S. suis* yielded negative results. These eight isolates may represent novel serotypes that should be further identified.

The majority of *S. suis* isolates from the farm-raised pigs tested in this study belonged to serotype 2; this result is consistent with that in a previous report (Wang et al., 2013a; Feng et al., 2014). All of these serotype 2 isolates were assigned into ST1 or ST28 clonal complex, indicating at least two parallel routes of clonal dissemination of these isolates. A total of 23 serotype 2 isolates (including 20 isolates from healthy pigs and three isolates from diseased pigs) were identified as ST7 strains. ST7 is a SLV of ST1 (allelic profile 1, 1, 1, 1, 1, 1, 1) with increased virulence (Ye et al., 2006); ST7 is also a member of the ST1 complex. ST7 strains that induced STSS and the presence of 89 K PaI caused the outbreaks of human infections in China in 1998 and 2005, respectively (Tang et al., 2006; Ye et al., 2006; Chen et al., 2007; Li et al., 2008). ST7 strains have been frequently isolated; as such, a routine survey of *S. suis* should be conducted in farm-raised pigs in northern China.

Piglets have been commonly used as experimental animal infection models to investigate *S. suis* pathogenicity. CD1 mice are highly sensitive to *S. suis*, and the virulence of *S. suis* in a CD1 mouse model is consistent with that in a piglet model (Quessy et al., 1995). *S. suis*-infected CD1 mice survive septicemia and later develop clinical signs of central nervous system disorders (such as locomotion problems, opisthotonus, and walking in circles), causing brain inflammation (Dominguez-Punaro et al., 2007). Therefore, the CD1 mouse is an effective experimental animal model to evaluate *S. suis* pathogenicity to cause shock and meningitis (Quessy et al., 1995; Dominguez-Punaro et al., 2007). Compared with piglets, CD1 mice exhibit several advantages, including convenient operation and low price.

In the present study, HWS9, HJMS, HDS, HJB, and HLB corresponding to ST417–ST421 were chosen to infect the CD1 mice. All of these strains caused septicemia and meningitis, leading to the death of mice. Among the four known major virulence loci (*mrp*, *sly*, *epf*, and 89 K PaI), only *epf* and *sly* were detected in HJMS and HDS; this result indicated the presence of additional virulence factors contributing to the observed virulence of these five strains in the CD1 mice.

The five tested strains could be divided into three groups based on mortality rates, the time at which clinical symptoms manifested, the degree of severity of clinical symptoms, and the time at which death occurred: high virulence group (strains HDS and HJB), moderate virulence group (HWS9), and low virulence group (HJMS and HLB). In the high virulence group, HJB caused super acute death as early as 1 day post-infection; by contrast, HDS induced death one or more days later. HJB was negative for *mrp*, *sly*, *epf*, and 89 K PaI. HJB caused acute fatal septicemia; bacteria *in vivo* migrated to different organs and caused tissue damage, but small bacterial loads were found in the liver and in the brain. Our results suggested that large amounts of bacterial endotoxin may be the major cause of the death of mice.

HLB yielded a final mouse mortality rate of 12.5%, displaying a very low level of lethality to the CD1 mouse. Nevertheless, this strain could still penetrate BBB of the CD1 mouse, causing suppurative encephalitis in the brain. This pathogen might induce a loss of blood-cerebrospinal fluid barrier function, an upregulation of proinflammatory mediator, or an increased leukocyte trafficking; each of these processes likely results in the heavy breakdown of BBB. By contrast, the four other novel ST strains (HDS, HJB, HWS9, and HJMS) did not cause suppurative encephalitis possibly because these strains induced a rapid progress of septicemia; thus, infected animals rapidly died without causing heavy brain damage in late disease phases.

In summary, 62 *S. suis* strains were isolated from healthy and diseased pigs in four provinces (Heilongjiang, Jilin, Liaoning, and Hebei) of northern China. Serotype 2 strains accounted for the majority of these strains, and these serotype 2 strains could be assigned into ST1 or ST28 clonal complex. Six novel STs, including ST417, ST418, ST419, ST420, ST421, and ST422, were found. Experimental infection with representative strains from ST417 to ST421 indicated that all of the tested strains could cause septicemia and meningitis in the CD1 mice but with remarkable differences in virulence; as a consequence, the mice died. These results illustrated that the zoonotic strains of *S. suis* in China are continually evolving; therefore, increased surveillance of *S. suis* in farm-raised pigs should be conducted.

## Conflict of Interest Statement

The authors declare that the research was conducted in the absence of any commercial or financial relationships that could be construed as a potential conflict of interest.

## Footnotes

1 http://ssuis.mlst.net

2 http://ssuis.mlst.net
